# Targeting BIP to induce Endoplasmic Reticulum stress and cancer cell death

**DOI:** 10.18632/oncoscience.330

**Published:** 2016-12-09

**Authors:** Michaël Cerezo, Rachid Benhida, Stéphane Rocchi

**Affiliations:** INSERM U1065 team 1, Université de Nice Cote d'Azur et Centre Méditerranéen de Médecine Moléculaire, Nice, France; ICN, UMR UNS-CNRS 7272, Nice, France

**Keywords:** BiP/GRP78, melanoma, Unfolded Protein Response, resistance, drug design

**Figure 1:**
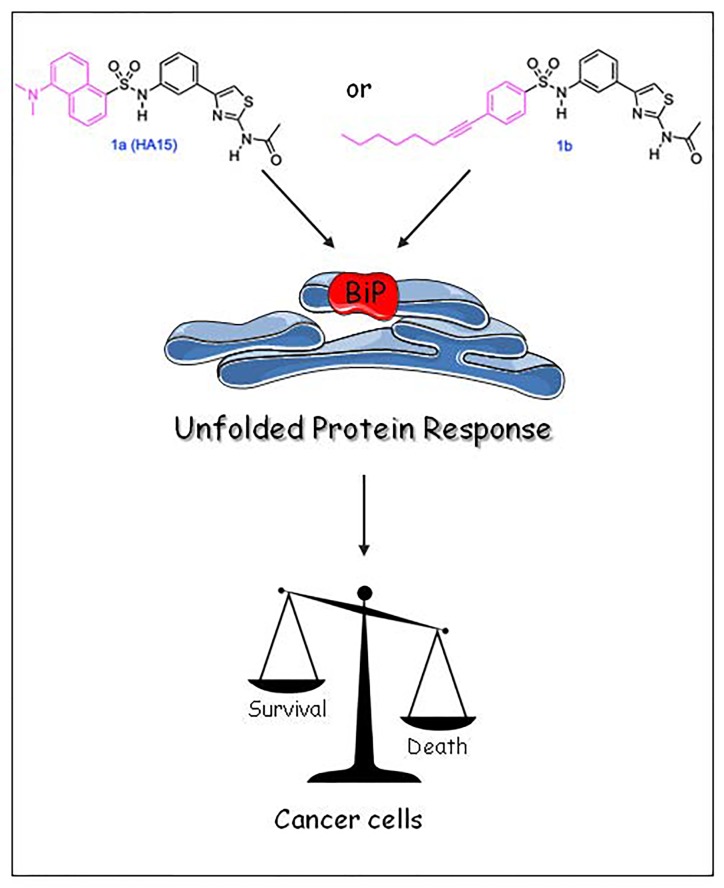
Effect of Thiazole Benzensulfonamides (TZB) on cancer cells.

Melanoma is the most aggressive form of skin cancer. Recently, significant progress has emerged with the development of new strategies in melanoma treatment. We currently have specific BRAF and MAP3K/MEK inhibitors. However, after a short period of remission, melanomas acquire drug resistance and recurrence of metastases is observed in almost all cases [1]. Second, immunotherapies targeted against CTLA4 and PD1, developed to reactivate the antitumor immune response of the patient, result in an objective and long-lasting response in only approximately 30% of patients [2]. Nevertheless, more than 50% of patients are currently in treatment failure. Therefore, identification of new potential targets is an urgent need to improved melanoma treatment. One promising strategy is the targeting of the Unfolded Protein Response pathway which appears as an emerging pathway to selectively target cancer cells. Indeed, neoplasic growth requires synthesis of lot of different proteins and Unfolded Protein Response is activated to deal with the high flux of proteins processed through the Endoplasmic Reticulum to maintained homeostasis [3]. Recently, we have identified a new molecules family, Thiazole Benzensulfonamides (TZB), whose HA15 (**1a**) molecule appears as the lead compound, that induce an elevated and maintained Endoplasmic Reticulum stress specifically in cancer cells without any adverse events in normal cells [4] (Figure 1). Briefly, HA15 induces death of all melanoma cells independently of their mutational status and melanoma cells freshly isolated from patients both sensitive or resistant to BRAF inhibitors. HA15 exhibited also a strong efficacy in xenograft mouse models performed with melanoma cells sensitive and resistant to BRAF inhibitors without any sign of toxicity. We next performed pan-genomic, proteomic and biochemical studies to decipher the signaling pathway, the mechanism of action and the target of the best candidate. We identified BIP, an endoplasmic reticulum protein, as the specific target of our compound. We demonstrated clearly that the interaction between our compound and BIP increases Endoplasmic Reticulum Stress and leads to melanoma cell death by concomitant induction of autophagy and apoptosis mechanisms. Overexpression of target BIP in various cancers is described, it is thus not surprising that this molecule was also found to be active against other liquid and solid tumors. Taken together, our data suggest HA15 has an important impact on inhibition of melanoma growth by targeting ER stress, and may therefore be developed for treatment of melanoma and other cancers.

Based on these strong data, we developed a lead optimization program in which two series of HA15 derivatives were synthesized that provided clear structure activity relationships. We then selected compound **1b** as a new optimized analogue of HA15 [5]. This compound was found to be ten-fold more active then the parent compound on various cancer cell lines including melanoma. Moreover, this optimized lead also exhibited strong activity against a set of cancer cells resistant to standard treatments and proved active in the low micromolar range. In addition, **1b** delayed significantly the tumor growth in vivo, with mice showing high tolerance for the compound. We also verified that this compound retains similar mode of action as HA15 by directly targeting BiP protein and by inducing cell death that involves a concomitant induction of autophagy and apoptosis mechanisms.

Furthermore, the notable in vivo efficacy and the absence of toxicity of HA15 and **1b** make this class of molecules particularly interesting as tools for chemical biology purposes for exploring apoptotic and autophagic signaling, as well as for clinical applications.

Interestingly, we have observed that TZB induce strong ER stress in cancer cell responsible to the cell death induction but only a moderate ER stress in normal cell without cell death induction. This differential effect could be due to a greater sensitivity of cancer cells to ER homeostasis perturbations as a result of elevated ER stress in cancer cells compared to normal cells. Indeed, the level of BiP, positively correlate with increased progression, tumour size and poor outcome for patients with melanoma [6].

Taking together, these studies highlight the key role of Unfolded Protein Response in melanoma and strengthen the idea that targeted protein chaperone like BiP could be useful alternative treatment in various spectrums of cancers, and particularly with this new class of compounds.
